# Effects of Smile Training on Gait Disturbance in Parkinson’s Disease Patient with Neuropsychiatric Symptoms: A Single Case Design

**DOI:** 10.1298/ptr.E10290

**Published:** 2024-09-11

**Authors:** Yumeka HARADA, Tatsuya IWABE, Keisuke OTA, Shinsuke HAMADA, Fumio MORIWAKA

**Affiliations:** 1 Department of Rehabilitation, Hokkaido Neurological Hospital, Japan; 2 Department of Physical Therapy School of Rehabilitation Sciences, Health Sciences University of Hokkaido, Japan; 3 Department of Neurology, Hokkaido Neurological Hospital, Japan

**Keywords:** Parkinson’s disease, Smile training, Gait disturbances, Depression, Anxiety

## Abstract

Objective: To verify the efficacy of smile training in improving gait disturbances in patients with Parkinson’s disease (PD) exhibiting neuropsychiatric symptoms. Methods: A single-case BAB design with three intervention periods (B1, A1, and B2) was used. During periods B1 and B2, 10 min of smile training (facial muscles training and positive thinking training) was performed before the usual exercise therapy. During the A1 period, the participant received only the usual exercise therapy. During the intervention period, the Timed Up and Go test (TUG) was performed daily in both directions. Tau-U was calculated to determine the effect size of the TUG test time and the number of steps taken during each period. Movement Disorder Society-Unified Parkinson’s Disease Rating Scale (MDS-UPDRS) Part III, Hospital Anxiety and Depression Scale (HADS), 10-meter walk at maximum speed, Berg Balance Scale, and Characterizing Freezing of Gait Questionnaire (C-FOGQ) were administered on the day before the start of the intervention and the last day of each period. Results: Comparisons of A1 to B2, TUG time, and the number of steps taken on both turns revealed large reductions (Tau-U ≥0.74, p <0.01). The 10-meter walk speed and MDS-UPDRS Part III bradykinesia scores improved, whereas the frequency of gait freezing on the C-FOGQ remained unchanged. The HADS scores did not show significant changes; however, the participant made more positive statements in his reflections. Conclusion: Smile training may be an effective intervention for improving gait and other motor symptoms in patients with PD.

## Introduction

Parkinson’s disease (PD) is a gradually progressive neurodegenerative disorder characterized by both motor and nonmotor difficulties, including neuropsychiatric symptoms^1)^. Depression is a prevalent and significant neuropsychiatric feature that affects a higher proportion of patients with PD than the general population^2)^. It is also the neuropsychiatric symptom that most strongly influences the quality of life of patients with PD^3)^. Anxiety is the second most common neuropsychiatric symptom of PD^4)^ which negatively affects quality of life^5)^. These neuropsychiatric symptoms are also common among Japanese patients, with a study reporting that 56%, 55%, and 41% of Japanese patients with PD experience depressive symptoms, anxiety symptoms, and both, respectively^6)^.

Neuropsychiatric symptoms have also been associated with gait disturbances in patients with PD^7^^–^^11)^. For instance, depressive symptoms are independent predictors of walking speed under both single- and dual-task conditions in patients with mild to moderate PD^7)^. Such gait disturbances have even been observed in patients with early-stage PD and mild depressive symptoms^8)^. Patients with PD and depression are at an increased risk of developing freezing of gait (FOG), a typical gait disorder in PD^9^^,^^10)^. Anxiety has also been linked to the development of FOG in patients with PD, although the evidence is inconclusive^11)^.

Although pharmacological treatment is common for neuropsychiatric symptoms in patients with PD^12)^, physical activity and cognitive behavioral interventions have also been proven to ameliorate depression and anxiety^13^^–^^17)^. Uchida and Arai^18)^ recently reported that a program combining voluntary facial muscles movements and an approach to fostering positive thinking (hereafter referred to as “smile training”) improved depressive symptoms and smile expressions in Japanese patients with PD. However, the potential of smile training in improving gait problems related to depression and anxiety in patients with PD has not been investigated.

Therefore, the possibility of pre-exercise therapy smile training enhancing walking ability in a hospitalized patient with PD, neuropsychiatric symptoms, and concurrent aggravated gait disturbances was examined in this single-case BAB design study.

## Methods

### Participant

The participant was a patient who had been admitted to our hospital and who met the following criteria: (1) diagnosed with PD by the attending physician, (2) aged ≥50 years at the time of consent, and (3) suspected of exhibiting neuropsychiatric symptoms based on observation. The exclusion criteria were as follows: (1) adjustments in antiparkinsonian or antidepressant medications during the study period, (2) severe cognitive impairment that made it difficult to understand the purpose of the study, (3) other Parkinsonian syndromes, and (4) deemed inappropriate for the study by the attending physician. The participant was a 76-year-old Japanese man with a 13-year history of PD characterized by an initial right lower limb tremor followed by progressive bradykinesia and a gait disturbance. The patient had begun medication for PD 9 years ago and had been hospitalized once or twice a year during this period. His admission at the time of the study was for a routine examination aimed at symptom improvement. However, his mood fluctuations had gradually worsened, as indicated by statements indicating increased symptoms of depression and anxiety (see Table 1) and diminished motivation for and participation in exercise activities. The Parkinson’s drugs that the participant was taking were Levodopa L100 (6 tablets per day), istradefylline 20 mg (two tablets per day), droxidopa 100 mg (nine tablets per day), zonisamide 25 mg (one tablet per day), and opicapone 25 mg (one tablet per day).

**Table 1.** The results of each assessment prior to the intervention, on the last day of each period, and at follow-up

	Pre-assessment	B1	A1	B2	2 weeks later	4 weeks later
10-meter walk test						
Walking speeds (m/s)	0.46	0.55	0.6	0.74	1.13	0.74
Cadence (steps/min)	124.5	92	117.8	114.5	128.8	114.7
MDS-UPDRS part Ⅲ						
Total	50	40	43	37	40	39
Tremor	7	7	7	7	7	7
Rigidity	9	9	9	9	9	9
Bradykinesia	21	13	16	10	13	12
Axial	13	11	11	11	11	11
BBS total	50	53	52	49	54	52
C-FOGQ 1-1	3–5	3–5	3–5	3–5	–	–
HADS-D	8	7	5	8	7	6
HADS-A	4	5	5	7	5	7
Subject’s introspection	• I just need to stretch today. • I’m off, so I’m stuck. • I may never walk again. • I can’t go home like this.	• Mouthing the words used in the positive thinking training. (I feel good today, etc.) • Maybe I can move today.	• I’ll try my best. ※ Continued statement in B1.	• I think I’ll make a move first. • Have a good feeling that one can do it. • I want to walk faster. • Let’s go for a walk.	–	–

MDS-UPDRS, Movement Disorder Society-Unified Parkinson’s Disease Rating Scale; BBS, Berg Balance Scale; C-FOGQ, Characterizing Freezing of Gait Questionnaire; HADS, Hospital Anxiety and Depression Scale

Ninety days after admission, his assessment results were as follows: Hoehn and Yahr severity classification was II on and IV off; The Movement Disorder Society-Unified Parkinson’s Disease Rating Scale (MDS-UPDRS) part III score was 50 (tremor, 7; rigidity, 9; bradykinesia, 21; and axial, 13); Berg Balance Scale (BBS) score was 50; Timed Up and Go test (TUG) was 23.7 s, with an increased number of steps during direction changes; and the Hospital Anxiety and Depression Scale (HADS) score was 12 (HADS-D = 8). In addition to the participant’s HADS-D score being above the cutoff score (suspected diagnosis at 8–10 points^19)^), his self-examination revealed numerous depressive symptoms. Although his statements also suggested possible anxiety, his questionnaire scores did not exceed the cutoff score for this diagnosis (suspected diagnosis at 8–10 points^19)^). No adjustments were made to any medications (including antiparkinsonian and psychotropic drugs) from this assessment until the end of the experiment. This case study complied with the Declaration of Helsinki and the Ethical Guidelines for Medical Research Involving Human Subjects and was approved by the Hokkaido Neurological Hospital Ethics Committee (FY2024NO2).

### Intervention

**Fig. 1.**
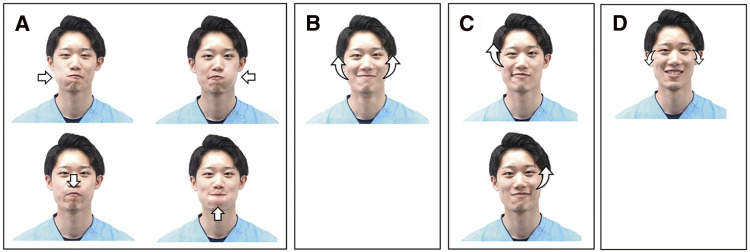
Facial muscles training (A) Orbicularis oris muscles exercise. An air ball was created on the right side of the mouth and moved to relax the orbicularis oris muscles. A mirror was used to ensure the proper movement of air toward the cheek, under the nose, and chin. (B) Lifting the corners of the mouth. The corners of the mouth were repeatedly raised and lowered, holding the raised position for at least 5 s. Proper elevation was ensured, and fingers were used for guidance as needed. (C) Vertical movement of the zygomaticus major. The zygomaticus major muscles was moved up and down. If difficult, a slight squint was tried for at least 5 s, and the fingers were used to check the muscles movement. (D) Lowering the eye corners. The patient focused on creating a natural smile by imagining pleasant thoughts and practicing smiling consistently. The patient was instructed to “remember to lower the corners of the eyes.”

A single-case BAB design was used with three intervention periods: B1, A1, and B2. In the BAB design, while immediate intervention would have been ethically preferable, to address the challenge of interpreting the B1 phase, outcome evaluations were conducted before the B1 phase and these results were set as the baseline. Each period lasted 40 min per day for 10 days (excluding Sundays), for a total of 30 days. During period B1, 10 min of smile training (expressive muscles training and positive thinking training) was administered before the usual exercise therapy (lower-limb stretching, resistance training, balance exercises, stair climbing, and walking exercises). During the study period, the duration of gait training in the usual exercise therapy intervention remained unchanged. In addition, inpatient rehabilitation included occupational and speech therapy, each conducted for 20–40 min per day, with no significant alterations in the intervention content. Furthermore, no self-practice was conducted throughout this time. Facial expression and positive thinking training were provided based on previous research^18)^. In the facial muscles training (Fig. 1), the participant performed four exercises while checking his facial expressions in the mirror: the orbicularis oris muscles exercise; lifting the corners of his mouth; vertical movement of the zygomaticus major; and lowering the corners of his eyes (exercises to create a natural smile). In the positive thinking training, he was asked to read aloud 10 words that promote positive thinking (“I feel good today,” “Things will get better,” “I am what I should be,” “I should smile,” “Greet as loudly as possible,” “I should have confidence,” “Things will work out,” “I could, I could,” “I am lucky,” “Thank you to everyone, thank you, thank you”) while visualizing the words. During period A1, only usual exercise therapy was administered. During period B2, the same interventions were performed as during period B1.

### Outcome measure and statistical analysis

During the intervention period, TUG scores (time and number of steps) for the main outcome were measured daily before the usual exercise therapy in the order of right and left turns. In the TUG, patients with PD have been reported to take more steps and to have a reduced step length compared to healthy older adults. This is likely to be influenced by disease severity and turn directions^20^^,^^21)^. Therefore, the number of steps was included as an outcome, and turns to both sides were assessed. The MDS-UPDRS part III, HADS, 10-meter walk at maximum speed, BBS, and Characterizing Freezing of Gait Questionnaire (C-FOGQ) were administered the day before the start of the intervention and on the last day of each period. HADS is a 14-item self-report measure of anxiety and depression that is widely used as a rapid screening tool in clinical settings around the world^19)^. C-FOGQ is an assessment tool designed to systematically evaluate the severity, frequency, and impact of FOG episodes in individuals with PD and related neurological conditions^22)^. After the end of the BAB period, follow-up assessments were performed at 2 and 4 weeks using the same measures as during the intervention period, except for the C-FOGQ. All the assessments were always carried out at the same time (“On” time). The participant’s reflective thoughts and perceptions, as articulated throughout the intervention, were documented in his medical records. These measurements were conducted by two physical therapists who were coresearchers in this case study. The slope and intercept of both the TUG test time and the number of steps for each distinct period were calculated using the least squares regression method. The Tau-U^23)^ was employed to ascertain the effect size pertaining to changes in TUG time and the number of steps. Tau-U is a metric used to assess the effect size of interventions in single-case designs, with values ranging from –1 to 1. Based on Rakap, Tau-U values are interpreted as follows: 0–0.65, 0.66–0.92, and 0.93–1.00 indicate weak or small, moderate to high, and strong or large effects, respectively^24)^. The significance level was established at 5%.

## Results

**Fig. 2.**
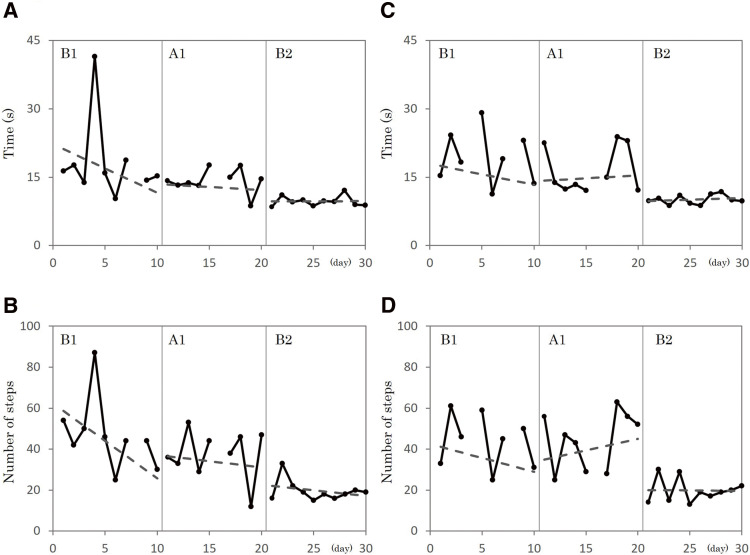
Changes in TUG scores in each period (A and B) Results of the TUG test during right turns. “A” represents the time duration (s), while “B” indicates the number of steps taken during the task. The dotted lines in each figure represent the slope and intercept derived using the least-squares method. Labels B1, A1, and B2 denote the intervention phases. The required time and number of steps decreased from A1 to B2. (C and D) Present TUG test outcomes during a left turn. “C” outlines the time duration (s), and “D” captures the number of steps. This trend was analogous to that of right turns, which exhibited more pronounced results. TUG, Timed Up and Go test

The TUG test scores (time and number of steps) measured during each period are shown in Figure 2. Despite the initial plan for the intervention to span 10 days in each period, interventions were not feasible on the eighth day of period B1 and the sixth day of period A1 because of the participant’s diminished blood pressure levels. Furthermore, the TUG assessment involving the left turn could not be conducted owing to a substantial decline in his motivation to engage in the exercise after the TUG right turn assessment on the fourth day of period B1. The TUG in the right turn, from period B1 to period A1, showed a slight reduction from 18.2 ± 9.07 s (mean ± standard deviation) to 14.2 ± 2.66 s (Tau-U, 0.43; p = 0.12) and a minor decrease in steps from 46.89 ± 17.59 to 37.56 ± 12.2 (Tau-U, 0.27; p = 0.33). The TUG in the right turn, from period A1 to period B2, revealed a very large time reduction from 14.2 ± 2.66 s to 9.71 ± 1.14 s (Tau-U, 0.8; p <0.01) and a large step decrease from 37.56 ± 12.2 to 19.6 ± 5.15 (Tau-U, 0.74; p <0.01). The slope of the TUG time in the right turn calculated by the least squares method showed a gradual slowing down: –1.06 in period B1; –0.13 in period A1; and 0.01 in period B2. Similarly, the TUG for left turns also demonstrated similar trends. From period B1 to period A1, there was a minor reduction in time from 19.2 ± 6.0 to 16.5 ± 5.1 s (Tau-U, 0.33; p = 0.25) and a slight decrease in steps from 43.33 ± 13.98 to 19.8 ± 5.83 (Tau-U, 0.01; p = 0.96). Furthermore, from period A1 to period B2, the left turn TUG time significantly decreased from 16.45 ± 5.09 to 10.09 ± 1.03 s (Tau-U, 1.00; p <0.01), and the number of steps reduced substantially from 44.33 ± 13.98 to 19.80 ± 5.83 (Tau-U, 0.87; p <0.01). The slopes of the TUG time in the left turn, calculated using the least-squares method, were –0.46, 0.13, and 0.08 in periods B1, A1, and B2, respectively, with a downward slope only in period B1. The TUG scores during the follow-up periods showed no significant changes from those recorded on the last day of period B2 (2 weeks: right turn 9.71 s/19 steps, left turn 8.91 s/21 steps; 4 weeks: right turn 9.93 s/23 steps, left turn 10.87 s/25 steps).

Table 1 shows the results of each assessment before the intervention, on the last day of each period, and at follow-up. Walking speed for 10-meter walking at maximal speed increased from 0.6 m/s at the end of period A1, to 0.74 m/s at the end of period B2, whereas cadence decreased from 117.8 rpm to 114.5 rpm. The MDS-UPDRS part III total score decreased by six points, from 43 points at the end of period A1 to 37 points at the end of period B2. The decreased score was caused by a reduction in bradykinesia. The total BBS score decreased from 52 at the end of period A1 to 49 at the end of period B2, indicating a slight improvement in balance capacity; however, this improvement was not sustained. The frequency of FOG in C-FOGQ 1-1 was three to five times per day and did not change during the intervention period. Neither HADS-D nor HADS-A showed a specific change in the trend from periods A1 to B2. On the other hand, regarding the participant’s introspection, positive statements such as “I will try to move,” increased in period B2, even on days when he felt he was not in good shape. In addition, he was frequently observed saying words and phrases used in the positive thinking training in period B2. No adverse events occurred during this intervention. In addition, the absence of negative comments or behaviors suggests that his psychological burden was minimal.

## Discussion

To our knowledge, this BAB single-case study is the first to examine whether pre-exercise smile training can improve walking ability in patients with PD, neuropsychiatric symptoms, and increased gait disturbance after hospitalization. Given that the content of parallel interventions and conventional exercise therapy showed no significant changes during the intervention period, the results indicate that pre-exercise smile training led to a marked improvement in gait speed and bradykinesia. However, its effects on neuropsychiatric symptoms and FOG require further scrutiny and empirical investigation. Nevertheless, the preliminary findings from this case study suggest that smile training is a practical and effective treatment approach for alleviating gait disturbances in patients with PD.

Smile training significantly enhanced walking capabilities, as evidenced by a statistically significant effect size and clinical relevance. The reduction in walking time and number of steps for the TUG test from period A1 to B2 period is likely to have been due to the smile training. The Tau-U values from A1 to B2 indicate that the intervention had a moderate to high effect on both TUG time and the number of steps for right and left turns^24)^. Specifically, Tau-U values for TUG time and the number of steps in right turns were 0.8 and 0.74, whereas in left turns, they were 1.00 and 0.87, respectively. These results suggest that the intervention significantly improved the participant’s walking speed and efficiency. While the improvement of 4.49 s for right turns did not surpass the minimal detectable change (MDC) threshold of 4.85 s in PD^25)^, the reduction of 6.36 s for left turns—where the less affected lower limb serves as the pivot—significantly exceeded this benchmark, indicating an enhancement in mobility that was not attributable to chance. Between phases B1 and B2, the mean walking time for both left and right turns decreased by more than 8 s, a change that surpassed the MDC threshold. In addition, the participant’s final TUG times dropped below the cutoff value of 11.5 s for fall risk on PD^26)^ and remained below this threshold throughout the follow-up period. An increase in 10-meter walk speed, surpassing the MDC of 0.25 m/s^27)^, was also observed. Therefore, the data suggest that as little as 10 min of smile training per day may have a beneficial impact on stabilizing gait, a finding that appears to be more than coincidental.

Focusing on the temporal changes in walking speed and the number of steps taken, it became evident that smile training immediately impacted these parameters and reduced the daily variability in this case. Specifically, the transition from phase A1 to B2 resulted in a marked decrease in both TUG test time and the number of steps taken, suggesting an immediate influence of smile training interventions on locomotor performance. Concurrently, the standard deviation, indicative of TUG time variability throughout the respective phases, demonstrated a reduction during the B1 phase, and the transition from A1 to B2 implied that smile training contributed to the stabilization and uniformity of walking patterns. The slope of the TUG test time in each period was markedly steeper in period B1 and gradually slower in periods A1 and B2. This may suggest that TUG-related motor learning occurred rapidly in phase B1 and then reached a saturation point during the subsequent phases. Smile training was also used in period B1, and the learning effect may have been facilitated by this intervention.

Smile training did not significantly alter the frequency of FOG as measured by the C-FOGQ; however, it may have contributed to the reduction in FOG episodes during the TUG test. The lack of measurable change in FOG frequency may be attributed to the lack of a specified time frame for recording FOG occurrences in the C-FOGQ, which may have led to inaccurate reporting of FOG frequency during each intervention phase. Importantly, a significant reduction was observed in the number of steps taken during the TUG test from period A1 to B2, suggesting a decrease in FOG episodes. Future research should consider using quantitative tools such as accelerometers for more precise measurements of FOG events^28)^ during TUG tasks, which were outside the scope of this case study.

In this instance, smile training did not affect the HADS score; however, it increased the number of positive statements made by the patient from periods A1 to B2. This is a contradictory finding, and the HADS assessment may not have accurately captured the mental and neurological symptoms at each stage. HADS is a questionnaire that generally surveys the participant’s “recent mood,” and in this study, the participant was not instructed to indicate the duration of the recent mood. In addition, HADS is a sensitive and specific measure of depressive symptoms in patients with PD; however, it is not as specific for anxiety indicators^1)^. Therefore, an improvement in neuropsychiatric symptoms might have improved, as observed from the patient’s reflections; however, this was not reflected in HADS. In addition, the increase in positive statements following smile training can be attributed to the patient’s inherently cheerful and humorous personality. Smile training may have helped the patient recall past positive emotions, leading to a positive shift in his self-reflections.

The results of this case study suggest that smile training may be a promising intervention for improving bradykinesia in patients with PD. A significant improvement was observed from the initial evaluation to the B2 final-day evaluation, and the effect was maintained at follow-up. Music therapy, also thought to improve emotional function, has been shown to improve bradykinesia^29)^. This finding suggests a relationship between emotional functioning and bradykinesia.

The mechanism by which smile training affects emotions and gait function can be considered as follows. The voluntary creation of a smile may form positive emotions based on the Facial Feedback Hypothesis^30)^. Furthermore, the activation of the ventromedial prefrontal cortex, insular cortex, and amygdala by positive words^31)^ suggests that the repeated expression of affirmative words may foster positive emotions. The facilitation of positive emotions can increase dopamine levels in the brain^32)^. Furthermore, the activity of the amygdala, which is involved in emotions, can impact motor performance^33)^. This influence on motor performance, associated with amygdala activity, may affect gait disturbances in PD through the basal ganglia circuits, including the limbic system^34)^. Therefore, in this case, the formation of positive emotions may have increased dopamine levels in the brain; regulated the activity of the limbic system, including the amygdala; and consequently improved gait speed and reduced the number of steps the participant required. However, further studies using neurophysiological methods are needed to elucidate the mechanisms involved.

This study has several limitations. First, it was limited to a single participant, making it difficult to generalize the findings. Second, the effects of the FOG, which affect the walking time of the TUG as a main outcome, could not be fully determined because it was not quantitatively evaluated. Third, the study did not include activities of daily living functional evaluations such as the functional independence measure, thus failing to examine changes in daily living activities. Fourth, the long-term effects of the intervention were not confirmed, limiting the understanding of its sustained efficacy. Finally, the results of the questionnaire surveys such as the C-FOGQ and HADS were difficult to interpret because they were not limited to episodes that occurred within the intervention period. Therefore, research with future applications of these evaluation tools should focus exclusively on episodes occurring within the intervention period. To address these limitations, it is, moreover, essential to involve a larger cohort of participants.

## Conclusion

This case study investigated whether pre-exercise smile training could improve a patient’s walking ability. The results showed that smile training improved the patients’ TUG time, number of steps, 10-meter walking speed, and bradykinesia. These improvements were maintained at follow-up. The participant’s introspections also suggested that smile training had a positive effect on his motivation for exercise. These findings provide preliminary evidence that pre-exercise smile training is an effective intervention for improving walking ability, specifically in terms of walking speed and the number of steps required in PD patients with neuropsychiatric symptoms and concurrent increased gait disturbances.

## Acknowledgments

We gratefully acknowledge Ms. Manami Takato’s significant contributions during the early stages of the study and the contributions of other colleagues.

## Funding

None.

## Conflicts of Interest

The authors have no conflicts of interest to declare.
